# Osmotic stress activates *nif* and *fix* genes and induces the *Rhizobium tropici* CIAT 899 Nod factor production via NodD2 by up-regulation of the *nodA2* operon and the *nodA3* gene

**DOI:** 10.1371/journal.pone.0213298

**Published:** 2019-03-27

**Authors:** Pablo del Cerro, Manuel Megías, Francisco Javier López-Baena, Antonio Gil-Serrano, Francisco Pérez-Montaño, Francisco Javier Ollero

**Affiliations:** 1 Departamento de Microbiología, Facultad de Biología, Universidad de Sevilla, Sevilla, Spain; 2 Departamento de Química Orgánica, Facultad de Química, Universidad de Sevilla, Sevilla, Spain; Universite Paris-Sud, FRANCE

## Abstract

The symbiosis between rhizobia and legumes is characterized by a complex molecular dialogue in which the bacterial NodD protein plays a major role due to its capacity to activate the expression of the nodulation genes in the presence of appropiate flavonoids. These genes are involved in the synthesis of molecules, the nodulation factors (NF), responsible for launching the nodulation process. *Rhizobium tropici* CIAT 899, a rhizobial strain that nodulates *Phaseolus vulgaris*, is characterized by its tolerance to multiple environmental stresses such as high temperatures, acidity or elevated osmolarity. This strain produces nodulation factors under saline stress and the same set of CIAT 899 nodulation genes activated by inducing flavonoids are also up-regulated in a process controlled by the NodD2 protein. In this paper, we have studied the effect of osmotic stress (high mannitol concentrations) on the *R*. *tropici* CIAT 899 transcriptomic response. In the same manner as with saline stress, the osmotic stress mediated NF production and export was controlled directly by NodD2. In contrast to previous reports, the *nodA2FE* operon and the *nodA3* and *nodD1* genes were up-regulated with mannitol, which correlated with an increase in the production of biologically active NF. Interestingly, in these conditions, this regulatory protein controlled not only the expression of nodulation genes but also the expression of other genes involved in protein folding and synthesis, motility, synthesis of polysaccharides and, surprinsingly, nitrogen fixation. Moreover, the non-metabolizable sugar dulcitol was also able to induce the NF production and the activation of *nod* genes in CIAT 899.

## Introduction

Legume plants can establish a symbiotic interaction with a group of soil bacteria, known as rhizobia, that fix atmospheric nitrogen in specialized root organs called nodules. This process requires a complex and evolved molecular dialogue between both organisms, which is initiated by the exudation of plant flavonoids [1]. These molecules are recognized by the NodD protein, a bacterial transcriptional regulator that binds to specific promoter sequences denominated *nod* boxes (NB), activating the expression of the nodulation (*nod*) genes. Proteins encoded by these genes are responsible for the synthesis and export of specific rhizobial lipochitooligosaccharides, also called Nod factors (NF) [2,3], whose recognition by the host plant triggers both rhizobial infection and initiation of nodule organogenesis [4]. Interestingly, in *Rhizobium tropici* CIAT 899 (hereafter CIAT 899), a broad host-range strain microsymbiont of *Phaseolus vulgaris* (common bean), the synthesis and export of NF is not only triggered by inducing flavonoids but also by acidity or high concentrations of salt [5–7]. The analysis of the CIAT 899 genome indicates that this bacterium harbours in the symbiotic plasmid three different *nodA* genes and five different *nodD* genes, which are responsible for the CIAT 899 capacity to produce a large variety of NF under different environmental conditions [8–11].

During their free-living stage in the soil, rhizobia are exposed to multiple physical stresses such as high temperatures, acidity or elevated osmolarity [12]. Interestingly, CIAT 899 is also characterized for tolerating all these stressing conditions [13]. Thereby, the CIAT 899 transcriptome in the presence of salt displays many differentialy expressed genes involved in osmotic-stress tolerance and adaptation [14]. Besides, under these stressing conditions, the same set of CIAT 899 nodulation genes activated by inducing flavonoids (*nodA1BCSUIJH* [under the control of *nod* box1, NB1], *nodA2hsnTnodFE* [NB2], *nodM* [NB3], *y4wEF* [NB4] and two genes with unknown functions [NB5]) are also up-regulated, suggesting that synthesis and export of NF occurs in the same manner. However, the salt-mediated production of NF in this bacterium is induced by NodD2, whereas flavonoid-induced synthesis is controlled by NodD1 [14–17]. This peculiar production of symbiotic molecules seems to be a strategy of CIAT 899 to ensure nodulation under osmotic stressing conditions [14].

In this paper, we have studied the *R*. *tropici* CIAT 899 transcriptomic response to the presence of high concentrations of mannitol to establish the similarities and differences between salt- and mannitol-mediated transcriptomic responses. Besides to changes in the expression values of genes related to osmotic-stress tolerance and mannitol metabolism, RNA-seq experiments indicated that not only some *nod* genes were up-regulated in the presence of mannitol but also operons implied in nitrogen fixation. Moreover, other non-metabolizable sugar (dulcitol) also induce the NF production and *nod* genes activation in CIAT 899. Finally, we determined that the activation of nodulation genes correlated with the overproduction of NF, which directly depended on the NodD2 protein.

## Materials and methods

### Bacterial strains and plasmids

Strains used in this study were grown at 28°C on tryptone yeast (TY) medium (0 mM mannitol) for RNA-seq studies [18], B^-^ minimal medium (55 mM mannitol) in NF-related experiments [19] or yeast extract mannitol (YM) medium (16,5 mM mannitol) in the ß-galactosidase assays [20], supplemented with the appropriate mannitol concentrations when necessary. *Escherichia coli* strains were cultured on LB medium [21] at 37°C. When required, the media were supplemented with the appropriate antibiotics as previously described [22].

The growing curves were obtained with a Sinergy HT microplate reader (BioTek, USA) and growing the bacteria for 72 h at 28°C with continuous orbital shaking. Measurements were made every 4 h.

In this work the wild strain *R*. *tropici* CIAT 899 [15] and their *nodD* mutant derivative strains were used: *nodD1* mutant [7], *nodD2* mutant [10], *nodD3*, *nodD4*, and *nodD5* mutants [11]. Plasmid pMP240 [23], which contains a transcriptional fusion between the *R*. *leguminosarum* biovar *viciae nodA* promoter and the *lacZ* gene, was transferred by conjugation to all these strains.

### RNA extraction and sequencing

*R*. *tropici* CIAT 899 and the Δ*nodD2* mutant were grown on 7 ml of TY medium, supplemented with 400 mM mannitol when necessary. Bacteria were incubated at an orbital shaker (180 rpm) for 72 h at 28°C. To ensure aeration, the 7 ml of TY cultures were placed in 50 ml Falcon tubes. Total RNA was isolated using a High Pure RNA Isolation Kit (Roche, Switzerland) according to the manufacturer’s protocol. Verification of the amount and quality of total RNA samples was carried out using a Nanodrop 1000 spectrophotometer (Thermo Scientific, USA) and a Qubit 2.0 Fluorometer (Invitrogen, USA). Two independent total RNA extractions were obtained for each condition and strain.

Ribosomal RNA was depleted using a MICROB Express Bacterial mRNA Purification kit (Ambion, USA), following the manufacturer´s protocol. Integrity and quality of the ribosomal depleted RNA was checked with an Agilent Bioanalyzer 2100 (Agilent Technologies, USA). RNA sequencing was carried out by Sistemas Genómicos (https://www.sistemasgenomicos.com/web_sg/) with the Next Generation Sequencing (NGS) platform Illumina with 100pb pair-end reads using the Illumina HiSeq 2000 sequencing instrument (Illumina, USA). Ribosomal-depleted samples were used to generate whole transcriptome libraries following the manufacturer's recommendations for sequencing on this NGS platform. Amplified cDNA quality was analyzed by the Bioanalyzer 2100 DNA 1000 kit (Agilent Technologies, USA) and quantified using the Qubit 2.0 Fluorometer (Invitrogen, USA). The RNA-seq data discussed in this work have been deposited in the Sequence Read Archive of NCBI under the accession numbers PRJNA470887, PRJNA326592 [17] and PRJNA305690 [14].

A total of 8 RNA-seq libraries corresponding to the wild-type and the *nodD2* mutant strain, both under control and mannitol 400 mM conditions were generated (two independent biological experiments for each condition). Quality control of each run, sample normalizations and statistical procedures were performed as previously described [14] (S1 File). Differentially expressed genes for each strain and condition were obtained by comparing with gene expression levels of the wild-type strain grown under control conditions (S2 and S3 Files). Data set was validated by *q*RT-PCR as previously described [14], obtaining in all cases positive correlation degrees in fold-change values between the *q*RT-PCR and the RNA-seq data (Table 1, S4 File).

**Table 1:** RNA-Seq data validation using *q*RT-PCR. Fold-change values were calculated using the ΔΔCt method and normalized to the reference gene 16S for 7 differentially expressed genes in the presence of mannitol 400 mM.

Gene name	ID / replicon	RNA-Seq		*q*RT-PCR	
		CIAT 899	*nodD2* mutant	CIAT 899	*nodD2* mutant
***mtlD***	YP_007335070.1 / chromosome	3.59	3.55	2.84±0.62	3.58±0.67
***mtlE***	YP_007335074.1 / chromosome	6.67	5.67	5.48±0.97	4.87±0.69
***fixC***	YP_007336103.1 / pRtrCIAT899b	5.65	5.42	3.86±0.21	7.26±1.23
***nodA2***	YP_007336039.1 / pRtrCIAT899b	2.57	1.57	2.78±0.33	1.16±0.16
***nodA3***	YP_007335966.1/ pRtrCIAT899b	3.59	2.13	3.04±0.86	2.07±0.24
***nodF***	YP_007335966.1 / pRtrCIAT899b	3.49	2.58	4.67±1.01	2.27±0.43
***nifH***	YP_007336128.1 / pRtrCIAT899b	11.90	9.06	12.25±2.43	8.66±1.86

### RNA-seq data analysis

**Fig 1:**
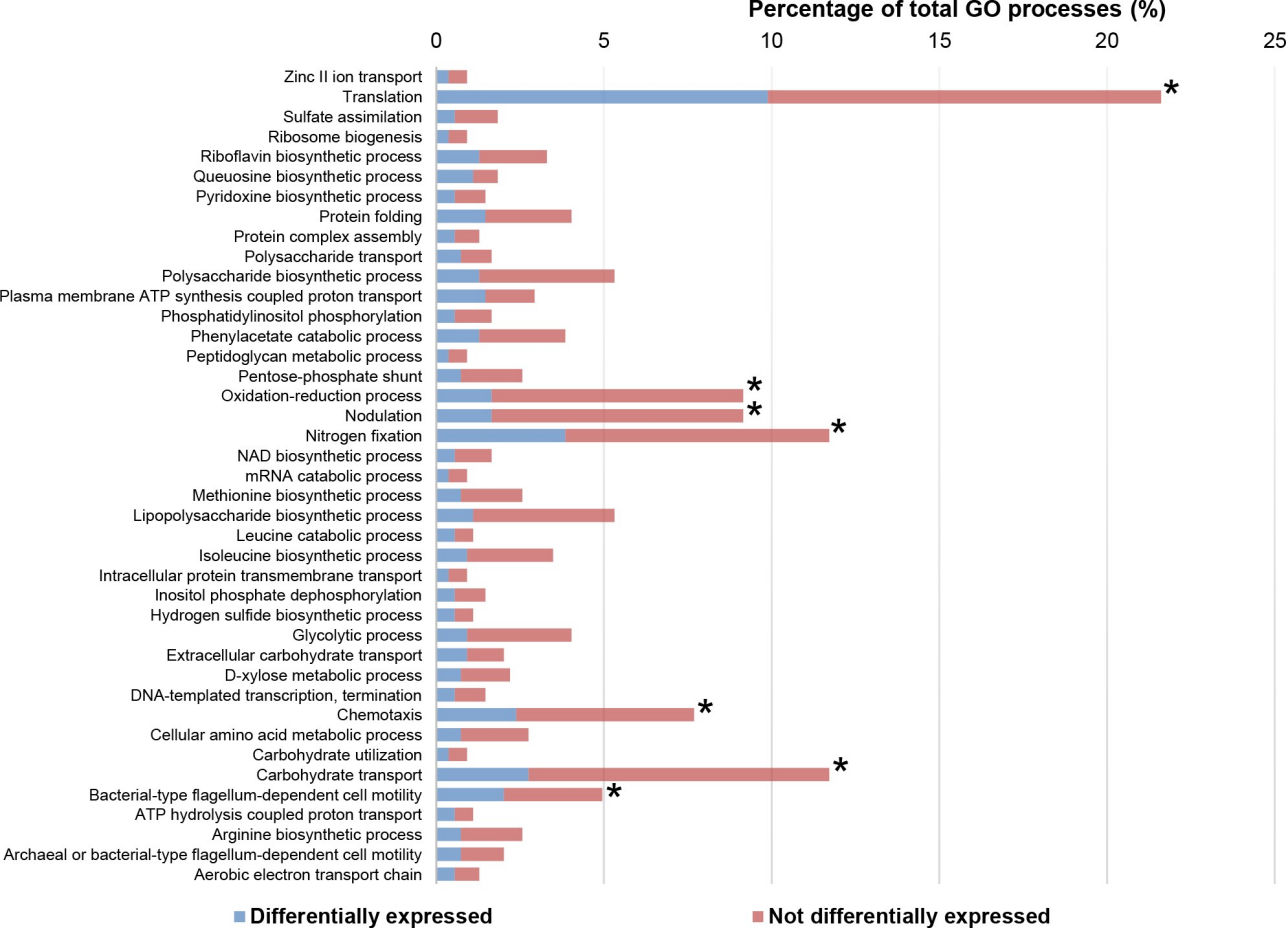
Percentage of genes from each Gene Ontology category differentially expressed with 400 mM mannitol. The percentage of genes shown corresponds to those genes present in the Gene Ontology database. Asteriks (*) indicate those categories with higher percentages.

In the primary analysis, a quality control of the raw data was carried out by fastq. In the secondary analysis, the initial whole transcriptome paired-end reads obtained from sequencing were mapped against the latest version of the *R*. *tropici* CIAT 899 genome (http://www.ncbi.nlm.nih.gov/genome/?term=Rhizobium_tropici_CIAT_899) using the mapping algorithm Bowtie2 v2.3.1 [24]. Low-quality reads were eliminated using Samtools [25,26] and Picard Tools (http://broadinstitute.github.io/picard/), remaining only high-quality reads. The genetic quantification was calculated using the htseq_count 0.6.1p1 method [27]. Gene differential expression and quantification were obtained by using DESeq2 [28]. In the tertiary anaylsis, the study of the concordance between the samples of the same conditions were made by a correlation study and Euclidean distance by using the statistic software R. To carry out the differential expression study among groups of samples Phyton and R software, and DESeq2 algorithm were used. Differentially expressed genes (DEG) in this study were established in those genes with a fold-change lower or higher than -2.5 or 2.5, respectively, with a *p* value adjusted to 0.05 by FDR [29]. Only the genes differentially expressed were considered for the biological processes represented from the Gene Ontology database (Fig 1).

### Quantification of nitrogenase activity

One colony of *R*. *tropici* CIAT 899 was grown for 72 h at 28°C in NFb [30] soft agar (0.4% agar) to simulate microaerobiosis conditions supplemented or not with 400 mM mannitol or dulcitol. After the incubation period, nitrogenase activity was quantified by acetylene reduction assays (ARA) as previously described [31]. *Azospirilum brasilense* Abv6 that also grows in 400 mM of mannitol, was used as positive control for ARA [32].

### Determination of the non-metabolizable sugar and culture media

The capacity of *R*. *tropici* CIAT 899 to use carbohydrates was studied by means of the API 50CH system (Biomérieux, France) according to the manufacturer’s instructions. YM and B^-^ media were used for β-galactosidase assays and Nod factor determination, respectively, in the presence of high concentrations of the non-metabolizable sugar. The YM and B^-^ media contained 16.5 mM and 55 mM of mannitol, respectively, sugar concentrations that ensure bacterial growth. The non-metabolizable sugar was added to culture media at the appropriated concentration.

### β-galactosidase assay

β-galactosidase activity assays with strains harbouring plasmid pMP240 were carried out as previously described [33] by using YM medium. Units of β-galactosidase activity were calculated according to Miller [34]. The experiments were repeated three times, with six replicates each time.

### RP-TLC analysis of NF and biological activity determination

Reversed-phase thin layer chromatography (RP-TLC) analyses were performed as previously described [19]. *R*. *tropici* CIAT 899 was grown on B^-^ minimal medium, supplemented when necessary with inducing molecules. For the NF radiolabeling, 0.2 μCi of N-acetyl-D-[1-^14^C]-glucosamine (specific activity 0.05 mCi) (Perkin Elmer) was used. Cultures of 1 mL were grown to the end of the exponential growth phase and the supernatant was extracted with water-saturated butanol. The butanol fraction was evaporated to dryness and the resulting powder dissolved in 40 μL of water-saturated butanol. This solution (10 μL) was applied to the TLC plate (RP-18F254S) (Merck, Germany), where the Nod factors were separated with 50% acetonitrile/H_2_O (vol/vol) as the mobile phase. TLC plates were exposed to a Fuji BAS-IIIs film for 10 days and the image was digitalized using the Phosphor-image system (Fujifilm, Japan).

NF were purified from 1 L of culture for each sample following the procedure previously described [35]. Purified NF were resuspended in 50 ml of acetonitrile 20% and 1 μL per mL of plant nutrient solution was added for biological activity assays. Thus, *P*. *vulgaris* Blue Bush Lake seeds were surface-sterilized and mounted in test tubes on a curled wire with the roots in 25 mL of Farhaeus medium [36]. Roots were protected from light and plants were grown for 10 days. Growth chamber conditions were 16 h at 26°C in the light and 8 h and 18°C in the dark, with 70% of humidity. To determine the presence of nodule primordia, roots were cleared with sodium hypochlorite and stained with methylene blue using the method of Truchet et al., 1989 [37]. Each experiment was repeated three times with six plants for each treatment.

### Nod factor determination by UHPLC-MS/MS

NF were purified as described above. Then, NF were analyzed with an Ultra High Preasure Liquid Chromatography (UHPLC) system consisting of a quaternary UHPLC Dionex Ultimate 3000 SD connected to a quadrupole-orbitrap Qexactive hybrid mass spectrometer (MS) (ThermoFisher Scientific, USA) with HESI ionization probe. Xcalibur software was used for instrument control and data acquisition. Separation was carried out using a Tracer Excel 120 ODSB C18 column (2.1 x 200 mm, 5 μm) (Teknokroma, Spain) at a flow rate of 0.3 ml/min. A binary gradient consisting of (A) water and (B) acetonitrile, both containing 0.1% formic acid, was used with the following elution profile: 50% B (5 min), linear gradient to 100% B (30 min), 100% B (2 min), linear up to 50% B (3 min) and finally 50% B (5 min). The injection volume was 20 μL. A Data Dependent Adquisition method (TOP5) was used in positive mode at resolution 70000 and 17500 at *m/z* 200 FWHM for Full Scan and Product Ion Scan, respectively. HESI source parameters were: spray voltage, 3.5 kV; S lens level, 50; capillary temperature, 320°C; sheath, auxiliary and sweep gas flow, 48, 11 and 2 respectively (arbitrary units); and probe heater temperature, 413°C. For data treatment, TraceFinder 3.3 software was used. The identification was made by comparing (maximum deviation of 5 ppm) the exact masses of the pseudomolecular ion and their fragment ions with the data contained in a LCO database with 1114 possible compounds. Isotopic pattern scores higher than 80% were also required.

## Results

### Differentially expressed genes and biological processes altered in the presence of mannitol

The most surprising trait of CIAT 899 relies on its ability to produce biologically active NF under salt-stressing conditions in a flavonoid-independent manner via NodD2 [7,17]. However, the question is whether NF production is induced only in the presence of salt, which generates both ionic and osmotic stresses, or whether it is also induced in the presence of other osmotic stresses such as high mannitol concentration. To shed light to these questions, we have analyzed the effect of high concentrations of mannitol on CIAT 899. First, to establish the appropriate concentration of solute, the growing curves of CIAT 899 in mannitol increasing conditions were obtained (TY medium supplemented with 0 mM to 1000 mM of mannitol). Concentrations higher than 500 mM of mannitol severely decreased bacterial growth-rate, whilst concentrations between 100 and 400 mM did not significantly affect growth (S5 File). For this reason, we established 400 mM of mannitol as the osmotic-stressing condition for further studies.

To study the effect of mannitol on the global gene expression of CIAT 899, transcriptomic assays were carried out. Thus, four RNA-seq libraries corresponding to CIAT 899 grown under control (TY medium) or mannitol 400 mM conditions were constructed. Two independent biological experiments were performed for each condition, being the general features of each run displayed in S1 File. Differentially expressed genes (DEG) in each condition are shown in S2 and S3 Files. Data set was validated by *q*RT-PCR experiments (Table 1, S4 File).

The *R*. *tropici* CIAT 899 genome harbours 6289 genes distributed among one chromosome (3672 CDS, CP004015.1) and three plasmids: pRtrCIAT899a/pA (212 CDS, CP004016.1), pRtrCIAT899b/pB/symbiotic plasmid (500 CDS, CP004017.1), and pRtrCIAT899c/pC (1905CDS, CP004018.1). The transcriptomic analysis showed a total of 743 DEG whose expression was activated or repressed 2.5-fold in the presence of 400 mM of mannitol (S2 File). Most of these genes were up-regulated (461 DEG, 62%). The replicon distribution revealed that most of these genes (468 DEG; 333 up-regulated and 135 down-regulated) were located in the chromosome, some of them in plasmid B (67 DEG; 66 up-regulated and 1 down-regulated) and the rest in plasmid C (36 DEG; 19 up-regulated and 17 down-regulated) (S6 File). Finally, to determine those functions actived or repressed by mannitol, a functional enrichment was performed to establish the significant over-represented biological processes using data available at the Uniprot database (Gene Ontology, GO). In the presence of mannitol, the biological processes statistically affected in CIAT 899 were translation, chemotaxis, carbohydrate transport, oxidation-reduction processes and, interestingly, nodulation and nitrogen fixation (Fig 1).

### High concentrations of mannitol activate both osmotic-stress tolerance and symbiotic genes

**Fig 2:**
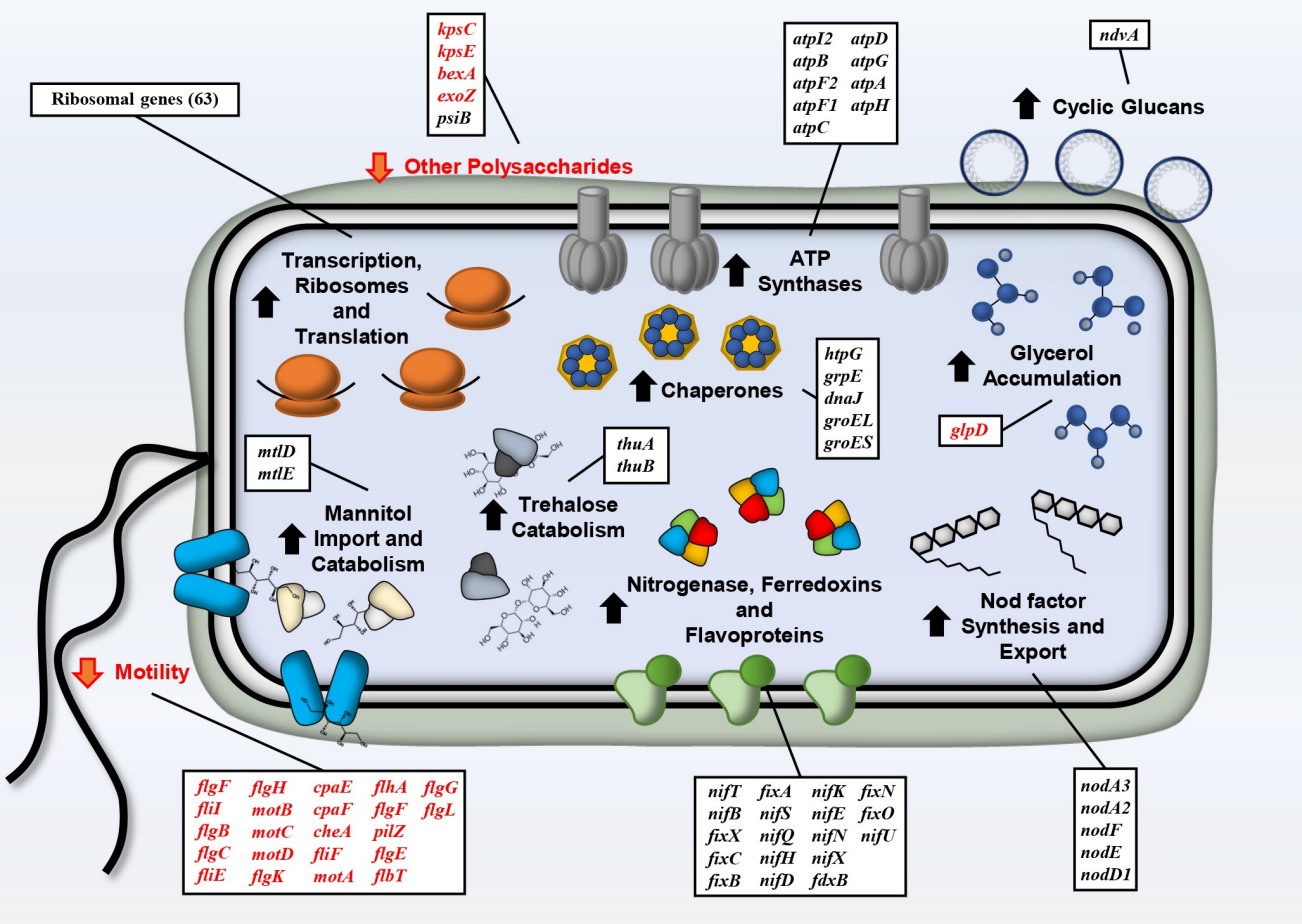
Genes and processes differentially regulated with 400 mM mannitol in *R*. *tropici* CIAT 899. Red labelled and black labelled gene names and arrows indicate down-regulation and up-regulation, respectively.

Regarding to DEG (Fig 2, Table 2), up to 64 genes coding for RNA polymerases (*rpo* genes), some components of the ribosomes (*rps*, *rpl* and *rpm* clusters), translation elongation factors (*fus*, *tuf*, *tsf* and *efp* genes) and 9 genes implied in the synthesis of ATP synthetase subunits (*atp* cluster) were up-regulated, indicating that CIAT 899 is activating the general pathways for protein synthesis and energy generation to overcome the osmotic stress. Interestingly, several genes coding for chaperones (*htpG*, *grpE*, *dnaJ*, *groEL* and *groES*) were highly activated under mannitol conditions, indicating that CIAT 899 is ensuring the suitable protein folding under this abiotic-stressing condition. As expected, several genes that encode proteins related to mannitol import and catabolism were activated in this condition (*mlt* genes). Furthermore, transcriptomic data indicated that CIAT 899 could be accumulating compatible osmolytes, since the *glpD* gene, which is responsible for glycerol degradation, was repressed in the presence of 400 mM mannitol (Table 2). However, *thuAB* genes involved in the trehalose catabolism were up-regulated in the presence of mannitol (Table 2). Interestingly, the analysis of DEG indicated that under mannitol conditions, CIAT 899 strongly repressed the *exo* and the *kps* genes involved in the synthesis of exopolysaccharide (EPS) and capsular polysaccharide (KPS), respectively, but genes involved in the biosynthesis of cyclin glucan (CG) were activated in the presence of mannitol (Table 2). On the other hand, up to 31 genes encoding proteins implied in the production and assembly of the flagellum were strongly down-regulated under mannitol conditions, indicating that this bacterium can reduce motility and chemotaxis when it detects high concentrations of this sugar. Interestingly, some nodulation genes such as *nodA2*, *nodF* and *nodE* (controlled by NB2), *nodA3* and *nodD1* were also up-regulated (ranging from 2.6- to 3.7-fold) in the presence of mannitol, which suggests that this bacterium may be synthesizing NF in the presence of another osmotic-stressing condition different to salt. However, induction levels of these nodulation genes were moderated in comparison with those values previously obtained upon salt induction (from 9.8- to 11.9-fold) [14]. Surprisingly, 19 genes coding for the enzymatic machinery (nitrogenase, ferredoxins and flavoproteins) implied in nitrogen fixation (*nif*, *fix* and *fdx* genes) were also strongly activated when CIAT 899 was grown under mannitol conditions (Fig 2) (Table 2). The up-regulation of this set of genes in the presence of mannitol did not correlate with real N_2_ fixation values in acetylene reduction experiments in microaerobiosis, indicating that other signals, molecules or conditions are required to perform this biological process outside of the legume nodule. *A*. *brasilense* Abv6, the positive control of ARA, was able to fix nitrogen in NFb medium supplemented or not with 400 mM mannitol or duciltol.

**Table 2:** Fold-change values of relevant genes from *Rhizobium tropici* CIAT 899 and its *nodD2* mutant derivative grown with 400 mM mannitol.

ID / Replicon	Gene name	Function	Biological Role	Fold-change wild-type^a^	Fold-change *nodD2* mutant^a^
**YP_007335070.1 / Chromosome**	*mtlD*	Mannitol 2-dehydrogenase	Mannitol catabolism	3.59	3.56
**YP_007335071.1/ Chromosome**	*-*	Sorbitol/mannitol ABC transporter, ATP-binding protein	Mannitol/Sorbitol importation	4.80	3.85
**YP_007335074.1 / Chromosome**	*mtlE*	Sorbitol/mannitol ABC transporter, substrate-binding protein	Mannitol/Sorbitol importation	6.67	5.68
**YP_007335245.1 / Chromosome**	*glpD*	Aerobic glycerol-3-phosphate dehydrogenase	Glycerol catabolism/osmolyte as compatible	-2.93	-3.7
**YP_007335966.1 / pRtrCIAT899b**	*nodA3*	Acyl transferase	Nod factor synthesis and nodulation	3.59	NDEG
**YP_007336039.1 / pRtrCIAT899b**	*nodA2*	Acyl transferase	Nod factor synthesis and nodulation	2.57	NDEG
**YP_007336041.1 / pRtrCIAT899b**	*nodF*	Acyl carrier protein	Nod factor synthesis and nodulation	3.49	NDEG
**YP_007336042.1 / pRtrCIAT899b**	*nodE*	β-ketoacyl synthase	Nod factor synthesis and nodulation	2.72	NDEG
**YP_007336077.1 / pRtrCIAT899b**	*nodD1*	Transcriptional regulator	Nod factor synthesis and nodulation	4.63	NDEG
**YP_007336097.1 / pRtrCIAT899b**	*nifT*	Nitrogen fixation protein	Nitrogen Fixation	5.04	NDEG
**YP_007336100.1 / pRtrCIAT899b**	*nifB*	Nitrogenase cofactor biosynthesis protein	Nitrogen Fixation	2.60	NDEG
**YP_007336102.1 / pRtrCIAT899b**	*fixX*	Ferredoxin protein	Nitrogen Fixation	4.26	3.52
**YP_007336103.1 / pRtrCIAT899b**	*fixC*	Electron transfer flavoprotein-quinone oxidoreductase	Nitrogen Fixation	5.65	5.42
**YP_007336104.1 / pRtrCIAT899b**	*fixB*	Electron transfer flavoprotein subunit alpha	Nitrogen Fixation	5.91	5.34
**YP_007336105.1 / pRtrCIAT899b**	*fixA*	Electron transfer flavoprotein subunit beta	Nitrogen Fixation	3.68	3.75
**YP_007336107.1 / pRtrCIAT899b**	*nifS*	Cysteine desulfurase	Nitrogen Fixation	3.23	NDEG
**YP_007336108.1 / pRtrCIAT899b**	*nifU*	Fe-S cluster assembly protein	Nitrogen Fixation	3.62	NDEG
**YP_007336110.1 / pRtrCIAT899b**	*nifQ*	Nitrogen fixation protein	Nitrogen Fixation	3.92	4.02
**YP_007336128.1 / pRtrCIAT899b**	*nifH*	Nitrogenase iron protein	Nitrogen Fixation	11.90	9.07
**YP_007336129.1 / pRtrCIAT899b**	*nifD*	Nitrogenase molybdenum-iron protein alpha chain	Nitrogen Fixation	14.00	10.77
**YP_007336130.1 / pRtrCIAT899b**	*nifK*	Nitrogenase molybdenum-iron protein beta chain	Nitrogen Fixation	10.71	8.41
**YP_007336131.1 / pRtrCIAT899b**	*nifE*	Nitrogenase iron-molybdenum cofactor biosynthesis protein	Nitrogen Fixation	13.79	10.49
**YP_007336132.1 / pRtrCIAT899b**	*nifN*	Nitrogenase iron-molybdenum cofactor biosynthesis protein	Nitrogen Fixation	10.48	8.03
**YP_007336133.1 / pRtrCIAT899b**	*nifX*	Nitrogen fixation protein	Nitrogen Fixation	8.74	5.52
**YP_007336134.1 / pRtrCIAT899b**	*-*	Nitrogen fixation protein	Nitrogen Fixation	9.39	7.15
**YP_007336136.1 / pRtrCIAT899b**	*fdxB*	Ferredoxin-3	Nitrogen Fixation	8.75	NDEG
**YP_007336050.1/ pRtrCIAT899b**	*fixO*	cbb3-type cytochrome c oxidase subunit II	Nitrogen Fixation	7.55	NDEG
**YP_007336049.1/ pRtrCIAT899b**	*fixN*	cbb3-type cytochrome c oxidase subunit I	Nitrogen Fixation	6.13	NDEG
**YP_007336979.1 / pRtrCIAT899c**	*htpG*	Chaperone	Protein folding in response to stress	5.20	3.72
**YP_007332415.1 / Chromosome**	*grpE*	Chaperone	Protein folding in response to stress	4.67	NDEG
**YP_007337672.1 / pRtrCIAT899c**	*dnaJ*	Chaperone	Protein folding in response to stress	2.62	NDEG
**YP_007332750.1 / Chromosome**	*groEL*	Chaperone	Protein folding in response to stress	6.27	6.89
**YP_007332751.1 / Chromosome**	*groES*	Chaperone	Protein folding in response to stress	5.66	6.58
**YP_007332230.1 / Chromosome**	*-*	Flp/Fap type IV pilin	Motility	-3.44	-5.20
**YP_007332235.1 / Chromosome**	*cpaE*	Pilus assembly protein	Motility	-3.45	NDEG
**YP_007332236.1 / Chromosome**	*cpaF*	Pilus assembly protein	Motility	-3.69	-3.87
**YP_007332594.1 / Chromosome**		Methyl-accepting chemotaxis protein	Motility	-6.76	-8.65
**YP_007332597.1 / Chromosome**	*cheA*	Chemotaxis protein	Motility	-5.34	-4.44
**YP_007332604.1 / Chromosome**	*fliF*	Flagellar M-ring protein	Motility	-3.50	-4.26
**YP_007332610.1 / Chromosome**	*-*	Flagellar motor switch protein N	Motility	-3.70	NDEG
**YP_007332612.1 / Chromosome**	*motA*	Chemotaxis protein (motility protein A)	Motility	-3.41	-3.18
**YP_007332613.1 / Chromosome**	*flgF*	Flagellar basal-body rod protein	Motility	-10.03	-12.05
**YP_007332614.1 / Chromosome**	*fliI*	Flagellar protein export ATPase	Motility	-7.69	-4.29
**YP_007332616.1 / Chromosome**	*flgB*	Flagellar basal body rod protein	Motility	-11.29	-6.48
**YP_007332617.1 / Chromosome**	*flgC*	Flagellar basal-body rod protein	Motility	-9.00	-7.83
**YP_007332618.1 / Chromosome**	*fliE*	Flagellar hook-basal body complex protein	Motility	-6.19	NDEG
**YP_007332619.1 / Chromosome**	*flgG*	Flagellar basal-body rod protein	Motility	-5.35	-3.71
**YP_007332623.1 / Chromosome**	*flgH*	Flagellar L-ring protein	Motility	-4.69	NDEG
**YP_007332627.1 / Chromosome**	*-*	Putative flagellin protein	Motility	-11.23	-10.62
**YP_007332628.1 / Chromosome**	*-*	Putative flagellin protein	Motility	-6.50	-7.50
**YP_007332634.1 / Chromosome**	*motB*	Chemotaxis motility protein	Motility	-5.08	-5.55
**YP_007332635.1 / Chromosome**	*motC*	Chemotaxis motility protein	Motility	-6.18	NDEG
**YP_007332636.1 / Chromosome**	*motD*	Chemotaxis motility protein	Motility	-7.22	-6.91
**YP_007332639.1 / Chromosome**	*flgK*	Flagellar hook-associated protein	Motility	-7.10	-7.05
**YP_007332640.1 / Chromosome**	*flgL*	Flagellar hook-associated protein	Motility	-6.11	-5.43
**YP_007332642.1 / Chromosome**	*flbT*	FlagellarFlbT family protein	Motility	-5.36	NDEG
**YP_007332645.1 / Chromosome**	*flhA*	Flagellar biosynthesis protein	Motility	-3.53	-2.72
**YP_007332822.1** **/ Chromosome**	*flgF*	flagellar basal-body rod protein	Motility	-3.09	NDEG
**YP_007332838.1/ Chromosome**	*-*	Methyl-accepting chemotaxis protein	Motility	-3.61	NDEG
**YP_007334811.1/ Chromosome**	*pilZ*	Type IV pilus assembly protein	Motility	-6.14	NDEG
**YP_007337874.1 pRtrCIAT899c**	*flgE*	Flagellar hook protein FlgE	Motility	-4.28	-5.87
**YP_007337988.1 pRtrCIAT899c**	*-*	Methyl-accepting chemotaxis protein	Motility	-6.56	-3.83
**YP_007333979.1 pRtrCIAT899c**	*-*	Methyl-accepting chemotaxis protein	Motility	-2.74	-2.66
**YP_007335234.1 Chromosome**	*-*	Methyl accepting chemotaxis protein	Motility	-6.28	NDEG
**YP_007332068.1 Chromosome**	*rpsA*	30S ribosomal protein S1	Protein synthesis	3.23	NDEG
**YP_007332085.1 Chromosome**	*rpsO*	30S ribosomal protein S15	Protein synthesis	5.64	5.57
**YP_007332296.1 Chromosome**	*rpmI*	50S ribosomal protein L35	Protein synthesis	14.76	13.00
**YP_007332297.1 Chromosome**	*rplT*	50S ribosomal protein L20	Protein synthesis	9.46	8.66
**YP_007332405.1 Chromosome**	*rpsT*	30S ribosomal protein S20	Protein synthesis	11.17	12.68
**YP_007333170.1 Chromosome**	*rplI*	50S ribosomal protein L9	Protein synthesis	5.71	4.36
**YP_007333172.1 Chromosome**	*rpsR*	30S ribosomal protein S18	Protein synthesis	7.74	6.25
**YP_007333173.1 Chromosome**	*rpsF*	30S ribosomal protein S6	Protein synthesis	14.37	10.06
**YP_007333286.1 Chromosome**	*rpsI*	30S ribosomal protein S9	Protein synthesis	6.19	4.44
**YP_007333287.1 Chromosome**	*rplM*	50S ribosomal protein L13	Protein synthesis	5.62	4.45
**YP_007333365.1 Chromosome**	*rpmG*	50S ribosomal protein L33	Protein synthesis	4.40	3.86
**YP_007333416.1 Chromosome**	*rplK*	50S ribosomal protein L11	Protein synthesis	5.10	5.06
**YP_007333417.1 Chromosome**	*rplA*	50S ribosomal protein L 1	Protein synthesis	6.51	6.03
**YP_007333418.1 Chromosome**	*rplJ*	50S ribosomal protein L10	Protein synthesis	7.69	7.70
**YP_007333419.1 Chromosome**	*rplL*	50S ribosomal protein L7/L 12	Protein synthesis	10.97	10.84
**YP_007333423.1 Chromosome**	*rpsL*	30S ribosomal protein S 12	Protein synthesis	21.82	18.09
**YP_007333424.1 Chromosome**	*rpsG*	30S ribosomal protein S7	Protein synthesis	16.69	12.51
**YP_007333427.1 Chromosome**	*rpsJ*	30S ribosomal protein S10	Protein synthesis	15.07	12.92
**YP_007333428.1 Chromosome**	*rplC*	50S ribosomal protein L3	Protein synthesis	13.92	11.65
**YP_007333429.1 Chromosome**	*rplD*	50S ribosomal protein L4/L1	Protein synthesis	18.07	14.96
**YP_007333430.1 Chromosome**	*rplW*	50S ribosomal protein L23	Protein synthesis	12.49	12.43
**YP_007333431.1 Chromosome**	*rplB*	50S ribosomal protein L23	Protein synthesis	12.19	10.22
**YP_007333432.1 Chromosome**	*rpsS*	30S ribosomal protein S19	Protein synthesis	10.13	8.64
**YP_007333433.1 Chromosome**	*rplV*	50S ribosomal protein L23	Protein synthesis	11.44	10.90
**YP_007333434.1 Chromosome**	*rpsC*	30S ribosomal protein S3	Protein synthesis	11.31	9.22
**YP_007333435.1 Chromosome**	*rplP*	50S ribosomal protein L16	Protein synthesis	12.64	10.19
**YP_007333436.1 Chromosome**	*rpmC*	50S ribosomal protein L29	Protein synthesis	10.08	8.88
**YP_007333437.1 Chromosome**	*rpsQ*	30S ribosomal protein S17	Protein synthesis	9.15	8.18
**YP_007333438.1 Chromosome**	*rplN*	50S ribosomal protein L14	Protein synthesis	12.35	13.05
**YP_007333439.1 Chromosome**	*rplX*	50S ribosomal protein L24	Protein synthesis	13.22	11.73
**YP_007333440.1 Chromosome**	*rplE*	50S ribosomal protein L5	Protein synthesis	14.14	12.68
**YP_007333441.1 Chromosome**	*rpsN*	30S ribosomal protein S14	Protein synthesis	13.12	11.95
**YP_007333442.1 Chromosome**	*rpsH*	30S ribosomal protein S8	Protein synthesis	11.76	11.02
**YP_007333443.1 Chromosome**	*rplF*	50S ribosomal protein L6	Protein synthesis	13.78	11.46
**YP_007333444.1 Chromosome**	*rplR*	50S ribosomal protein L18	Protein synthesis	12.44	11.99
**YP_007333445.1 Chromosome**	*rpsE*	30S ribosomal protein S5	Protein synthesis	16.91	15.70
**YP_007333446.1 Chromosome**	*rpmD*	50S ribosomal protein L30	Protein synthesis	6.89	7.33
**YP_007333447.1 Chromosome**	*rplO*	50S ribosomal protein L15	Protein synthesis	10.06	8.90
**YP_007333450.1 Chromosome**	*rpsM*	30S ribosomal protein S13	Protein synthesis	8.97	9.34
**YP_007333451.1 Chromosome**	*rpsK*	30S ribosomal protein S11	Protein synthesis	13.16	11.73
**YP_007333453.1 Chromosome**	*rplQ*	50S ribosomal protein L17	Protein synthesis	8.66	8.23
**YP_007333632.1 Chromosome**	*rpsB*	30S ribosomal protein S2	Protein synthesis	4.56	4.79
**YP_007333904.1 Chromosome**	*rpsD*	30S ribosomal protein S4	Protein synthesis	10.37	9.68
**YP_007334465.1 Chromosome**	*rplY*	50S ribosomal protein L25, Ctc-form	Protein synthesis	8.51	8.18
**YP_007334889.1 Chromosome**	*rpmE*	50S ribosomal protein L31	Protein synthesis	14.18	10.95
**YP_007334904.1 Chromosome**	*rpmJ*	50S ribosomal protein L36	Protein synthesis	8.91	6.00
**YP_007334990.1 Chromosome**	*rpsU*	30S ribosomal protein S21	Protein synthesis	4.10	4.58
**YP_007335204.1 Chromosome**	*rpmB*	50S ribosomal protein L28	Protein synthesis	7.06	6.65
**YP_007335344.1 Chromosome**	*rpsP*	30S ribosomal protein S16	Protein synthesis	3.85	3.24
**YP_007335345.1 Chromosome**	*rimM*	16S rRNA processing protein RimM	Protein synthesis	2.80	2.91
**YP_007335348.1 Chromosome**	*rplS*	50S ribosomal protein L19	Protein synthesis	8.21	8.54
**YP_007335513.1 Chromosome**	*rpmF*	50S ribosomal protein L32	Protein synthesis	12.71	10.94
**YP_007335588.1 Chromosome**	*rplU*	50S ribosomal protein L21	Protein synthesis	5.66	5.86
**YP_007335589.1 Chromosome**	*rpmA*	50S ribosomal protein L27	Protein synthesis	6.07	5.76
**YP_007333425.1 Chromosome**	*fusA*	Translation elongation factor G	Protein synthesis	13.04	10.99
**YP_007333426.1 Chromosome**	*tuf*	Elongation factor Tu	Protein synthesis	10.93	8.56
**YP_007335088.1 Chromosome**	*infA*	Translation initiation factor IF_1	Protein synthesis	7.07	5.62
**YP_007333633.1 Chromosome**	*tsf*	Translation elongation factor Ts	Protein synthesis	5.71	5.69
**YP_00733479.1 Chromosome**	*efp*	Translation elongation factor P	Protein synthesis	5.51	NDEG
**YP_007333111.1 Chromosome**	*rpoZ*	DNA-directed RNA polymerase subunit omega	Transcription	3.82	3.54
**YP_007333420.1 Chromosome**	*rpoB*	DNA-directed RNA polymerase, beta subunit	Transcription	4.88	4.38
**YP_007333421.1 Chromosome**		DNA-directed RNA polymerase, beta' subunit	Transcription	3.80	3.70
**YP_007333452.1 Chromosome**	*rpoA*	DNA-directed RNA polymerase, alpha subunit	Transcription	8.56	8.35
**YP_007336111.1 pRtrCIAT899b**	*rpoN*	RNA polymerase sigma-54 factor	Transcription	3.95	3.19
**YP_007332788.1 Chromosome**	*atpI2*	ATP synthase protein, subunit I	ATP synthesis	3.19	3.68
**YP_007332789.1 Chromosome**	*atpB*	ATP synthase F0, A subunit	ATP synthesis	3.06	2.93
**YP_007332791.1 Chromosome**	*atpF*	ATP synthase B' chain (Subunit II)	ATP synthesis	4.46	4.08
**YP_007332792.1 Chromosome**	*atpF*	ATP synthase B chain (Subunit I)	ATP synthesis	3.90	3.40
**YP_007335253.1 Chromosome**	*atpC*	ATP synthase F1, epsilon subunit	ATP synthesis	4.31	3.45
**YP_007335254.1 Chromosome**	*atpD*	ATP synthase F1, beta subunit	ATP synthesis	3.96	3.37
**YP_007335255.1 Chromosome**	*atpG*	ATP synthase F1, gamma subunit	ATP synthesis	3.34	3.38
**YP_007335256.1 Chromosome**	*atpA*	ATP synthase F1, alpha subunit	ATP synthesis	3.78	3.51
**YP_007335257.1 Chromosome**	*atpH*	ATP synthase F1, delta subunit	ATP synthesis	3.31	4.40
**YP_007332861.1 Chromosome**	*-*	Polysaccharide deacetylase	Polysaccharide synthesis	-3.30	-3.01
**YP_007333276.1 Chromosome**	*-*	Exopolysaccharide transport	EPS transport	-2.73	-3.64
**YP_007335159.1 Chromosome**	*-*	Polysaccharide biosynthesis protein	Polysaccharide synthesis	-4.01	NDEG
**YP_007335531.1 Chromosome**	*ndvA*	Cyclic beta-1,2-glucan exporter	Cyclic-glucan transport	4.24	NDEG
**YP_007337335.1 pRtrCIAT899c**	*kpsE*	KPS polysaccharide exportation	KPS transport	-10.59	-8.51
**YP_007337336.1 pRtrCIAT899c**	*-*	KPS polysaccharide exportation	KPS transport	-13.71	-4.87
**YP_007337340.1 pRtrCIAT899c**	*kpsC*	KPS polysaccharide exportation	KPS transport	-6.84	-3.99
**YP_007337343.1 pRtrCIAT899c**	*kpsE*	KPS polysaccharide exportation	KPS transport	-7.62	-6.14
**YP_007337344.1 pRtrCIAT899c**	*bexA*	ATP-binding protein	Polysaccharide transport	-6.40	-6.38
**YP_007337347.1 pRtrCIAT899c**	*-*	Polysaccharide export protein	Polysaccharide transport	-12.45	-6.32
**YP_007337762.1 pRtrCIAT899c**	*exoX*	Exopolysaccharide production repressor protein	EPS synthesis repressor	-3.25	-5.08
**YP_007337766.1 pRtrCIAT899c**	*exoZ*	Succinoglycan biosynthesis acetyltransferase	EPS synthesis	-2.86	NDEG
**YP_007336072.1 pRtrCIAT899b**	*psiB*	Putative polysaccharide inhibition protein	Polysaccharide synthesis repressor	3.05	NDEG
**YP_007334642.1 Chromosome**	*thuA*	putative trehalose oxidoreductase	Trehalose catabolism	6.05	5.13
**YP_007334643.1 Chromosome**	*thuB*	trehalose utilization protein	Trehalose catabolism	6.53	5.82

^**a**^ Fold induction with respect to CIAT 899 uninduced cultures.

NDEG: non-differentially expressed genes.

Light grey: DEG controlled by NodD2.

### The up-regulation of nodulation genes in the presence of mannitol is controlled by NodD2

**Fig 3:**
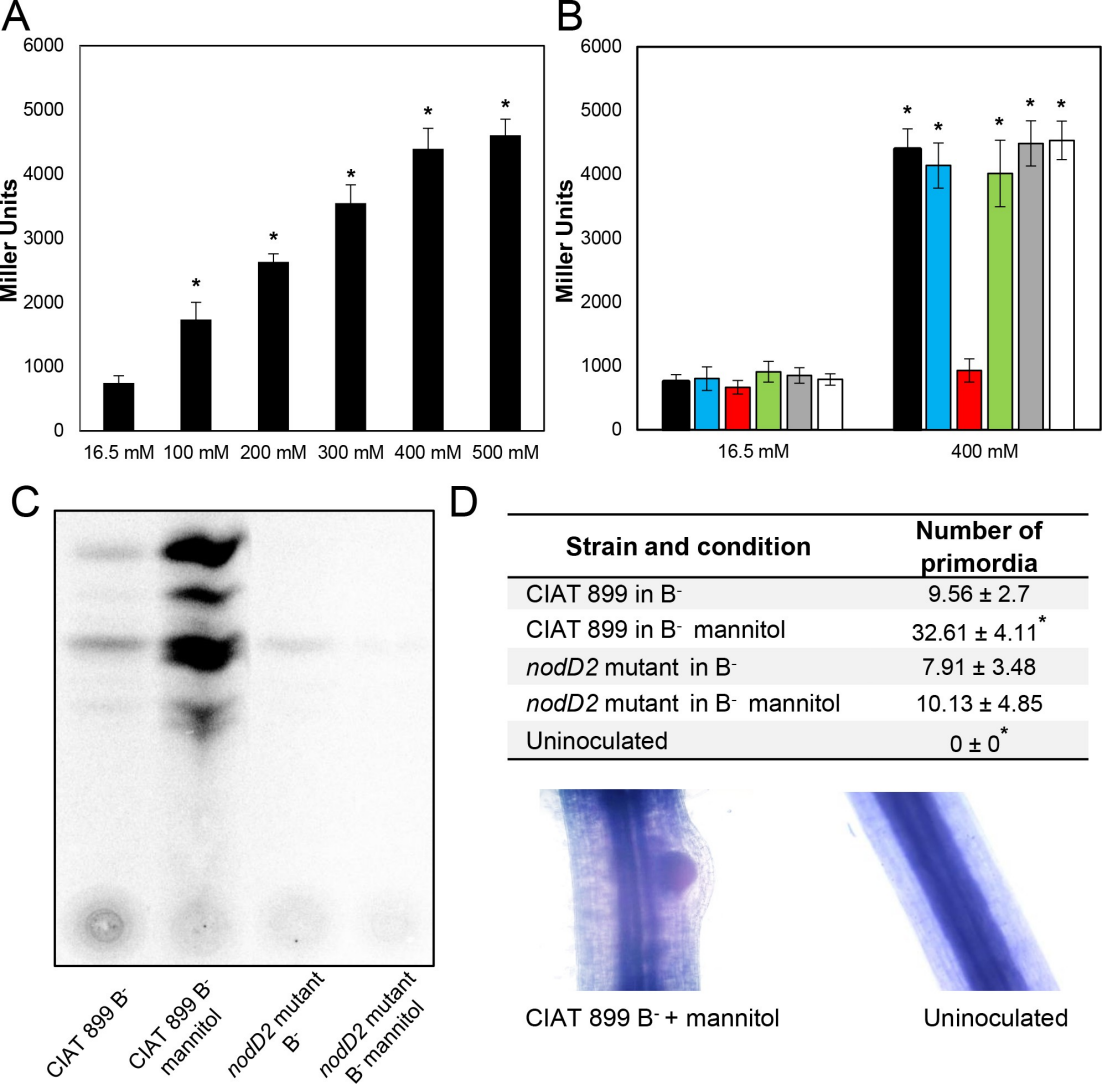
**A.** β-galactosidase activity of *R*. *tropici* CIAT 899 carrying plasmid pMP240 under different mannitol concentrations (55–500 mM). **B.** β-galactosidase activity of *R*. *tropici* CIAT 899 and its *nodD* mutant derivative strains carrying plasmid pMP240 under 400 mM mannitol. Black, blue, red, green, gray and white bars represent β-galactosidase activity levels of the wild-type, *nodD1* mutant, *nodD2* mutant, *nodD3* mutant, *nodD4* mutant and *nodD5* mutant strains, respectively. Expression data were individually compared with the expression without inducing molecules of the wild-type strain using the Mann-Whitney non-parametrical test. The asterisks (*) indicate a significant difference at the level α = 5%. **C.** Thin-layer chromatography analysis of Nod factors produced by *R*. *tropici* CIAT 899 and the *nodD2* mutant grown under control and 400 mM mannitol conditions. **D**. Biological activity assay of the NF purified from the wild-type and the *nodD2* mutant strain applied to common bean roots. The number of nodule primordia was individually compared to those primordia formed by the wild-type strain using the Mann-Whitney non-parametrical test. The asterisks (*) indicate a significant difference at the level α = 5%.

Our previous works have demonstrated that CIAT 899 nodulation genes (*nodA1BCSUIJH* [NB1], *nodA2hsnTnodFE* [NB2], and *nodM* [NB3]) are induced in the presence of apigenin and salt (NaCl 300 mM), and the regulation of the expression of these genes is addressed by NodD1 and NodD2, respectively [14,17]. Interestingly, in this RNA-seq study, we observed that among genes involved in nodulation, only those controlled by NB2 were significatly up-regulated in the presence of mannitol (S3 File). In order to elucidate which of the five *nodD* genes present in the CIAT 899 genome is involved in the activation of the *nod* genes in the presence of mannitol, we first performed different β-galactosidase activity assays in YM medium by using a CIAT 899 strain harbouring plasmid pMP240, which harbours the conserved *nodA* promoter of *R*. *leguminosarum* bv. *viciae* fused to the *lacZ* gene. As expected, the activation level of the *nodA* promoter region at 400 mM of mannitol (condition selected in this study for the RNA-seq) increased almost 6-fold when compared to control conditions (16.5 mM mannitol), supporting data obtained by the RNA-seq assay (Fig 3A). Similar ß-galactosidase activity values were reported when inducing with salt [17]. To determine whether one or more of the five CIAT 899 NodD proteins were responsible for the up-regulation of the nodulation genes with mannitol, we performed the same experiments in the five different *nodD* mutant backgrounds, all of them harbouring plasmid pMP240. Results showed that the activation of the nodulation genes in CIAT 899 was mediated by NodD2, since only in the *nodD2* mutant background the *nod* gene induction values in the presence of mannitol remained as in uninduced cultures (Fig 3B). We also performed a global transcriptomic assay in the presence and absence of mannitol in TY medium in the *nodD2* mutant background. RNA-seq data indicated that all the DEG involved in NF synthesis and export previously mentioned were not differentially expressed in this mutant grown with mannitol, supporting previous β-galactosidase activity results (Table 2). Interestingly, some other DEG identified in the wild-type RNA-seq experiments seemed to be directly or indirectly regulated by NodD2, since their expressions were altered in the *nodD2* mutant. Among them, we found some but not all the genes implied in nitrogen fixation, protein folding and synthesis, motility and synthesis of polysaccharides (Table 2).

### Nod factors synthesized in the presence of mannitol are biologically active

According to transcriptomic data and β-galactosidase assays, the biosynthesis of NF could be induced by CIAT 899 in response to the osmotic stress generated when this strain grows in the presence of 400 mM of mannitol. To determine whether these transcriptomic and β-galactosidase experiments correlated with a real NF production, we performed thin layer chromatography (TLC) in B^-^ medium to obtain the NF profiles. Results indicated that the presence of 400 mM mannitol strongly induced NF production in the wild-type strain and, as expected, this overproduction of NF was not detected for the *nodD2* mutant strain (Fig 3C), supporting previous experiments and confirming that the NodD2 protein is directly responsible for the synthesis and export of NF under high mannitol concentration conditions. Besides, to determine the biological activity of these molecules, we analysed the effect of mannitol-induced NF on *P*. *vulgaris* roots. Interestingly, we observed that even in the case of uninduced CIAT 899 cultures (B^-^ medium, 55 mM mannitol), the purified extract slightly induced the formation of primordia as previously observed [17]. However, a significant increase (3-fold) in the number of primordia was observed when NF purified from the wild-type strain grown in B^-^ medium with 400 mM of mannitol were added (Fig 3D). In the case of the biological activity of NF obtained from the *nodD2* mutant, the number of induced primordia was similar to those obtained by CIAT 899 in control conditions, indicating that the NF induced by mannitol are biologically active. Lastly, the NF produced by the wild-type and the *nodD2* mutant strain in B- medium supplemented or not up to 400 mM mannitol were identificated by UHPLC-MS/MS (Table 3). As expected, 36 different NF were found in supernatants of CIAT 899 cultures supplemented up to mannitol 400 mM, 25 of them were also produced in the presence of salt [10,11]. Only 6 and 4 nodulation factors were found in the wild-type cultures under control conditions or in both conditions in the *nodD2* mutant strain, respectively, confirming that synthesis and export of these symbiotic molecules is controlled by NodD2. Basal production of NF in *R*. *tropici* CIAT 899 has been previously reported [10,11], and could be responsible for the formation of nodule primordia even in the absence of inducing molecules [17] (Fig 3D).

**Table 3:** Structures of Nod Factor synthesized in the presence or absence of 400 mM mannitol by *Rhizobium tropici* CIAT 899 and its *nodD2* mutant derivative.

NF structure^a^	CIAT 899^b^	CIAT 899 in B^-^ mannitol^b^	*nodD2* mutant^b^	*nodD2* mutant in B^-^ mannitol^b^
**III (C_16:0_)**	-	+*	-	-
**III (C_16:0_, NMe)**	-	+*	-	-
**III (C_18:0_)**	-	+	-	-
**III (C_18:1_)**	-	+*	-	-
**III (C_18:1_, NMe)**	-	+*	-	-
**III-Hex (C_18:1_)**	-	+	-	-
**III-Hex (C_18:1_, NMe)**	-	+	-	-
**IV (C_14:0_)**	-	+*	-	-
**IV (C_14:0_, NMe)**	-	+*	-	-
**IV (C_16:0_)**	-	+*	-	-
**IV (C_16:0_, NMe)**	-	+*	-	-
**IV (C_16:1_)**	-	+*	-	-
**IV (C_18:0_)**	-	+*	-	-
**IV (C_18:0_, NMe)**	-	+*	-	-
**IV (C_18:1_)**	+	+*	+	+
**IV (C_18:1_, NMe)**	-	+*	-	-
**IV (C_18:1_, NMe, S)**	-	+*	-	-
**IV (C_20:1_)**	-	+	-	-
**IV-Hex (C_16:0_, NMe)**	-	+*	-	-
**IV-Hex (C_18:0_)**	-	+	-	-
**IV-Hex (C_18:0_, NMe)**	-	+	-	-
**IV-Hex (C_18:1_)**	-	+*	+	+
**V (C_16:0_)**	-	+*	-	-
**V (C_18:1_, NMe) dNAc^c^**	+	+	-	-
**V (C_18:1_, Cb) dNAc^c^**	-	+	-	-
**V (C_18:1_, NMe) dNAc^d^**	+	+	-	-
**V (C_16:0_, NMe)**	-	+*	-	-
**V (C_18:1_)**	+	+*	+	+
**V (C_18:0_)**	-	+*	-	-
**V (C_18:0_, S)**	-	+	-	-
**V (C_18:1_, NMe)**	+	+*	+	+
**V (C_18:1_, S)**	-	+*	-	-
**V (C_18:1_, NMe, S)**	+	+*	-	-
**V (C_18:0_, NMe, S)**	-	+*	-	-
**V (C_20:1_, NMe)**	-	+	-	-
**V (C_20:1_, NMe, S)**	-	+*	-	-
**Total**	6	36 (25*)	4	4

**a.** NF structures are represented following the convention in Spaink, 1992 [48] that indicates the number of GlcNAc residues in the backbone (Roman numeral), the length and degree of unsaturation of the fatty acyl chain, and the other substituents, which are listed in the order in which they appear, moving clockwise from the fatty acid. Hex, Hexose; NMe, N-methyl group at glucosamine non-reducing residue; S, sulfate group at reducing glucosamine residue; Cb, carbamoyl group.

**b.** Symbol: + = detected;— = not detected.

**c.** Nod Factor deacetylated at glucosamine residue number 3.

**d.** Nod Factor deacetylated at glucosamine residue number 4.

* NF also synthetized in the presence of salt [10, 11].

### *nod* gene expression and Nod factor production are also enhanced in the presence of a non-metabolizable sugar

In order to unequivocally assert that the osmotic stress caused by high mannitol concentration is responsible for the synthesis and export of Nod factors in *R*. *tropici* CIAT 899, we wondered whether this biological process is also launched in the presence of a non-metabolizable sugar. For this purpose, by using the API 50CH system, it was determined that CIAT 899 is unable to use and grow using sugars as unique carbon source, such as dulcitol, amydaline, xylitol, arabitol, inuline or raffinose. For further experiments we selected dulcitol as non-metabolizable sugar, since this molecule is an isomeric form of mannitol, being both polyol sugars that have the same molecular weight (182.172 g/l) and empirical formula of C_6_H_14_O_6_. As expected, in the presence of increasing amounts of dulcitol, *nod* gene expression was enhanced, reaching at 383.5 mM dulcitol (+ mannitol 16.5 mM) similar values of ß-galactosidase activity than those obtained with 400 mM mannitol (Figure A in S7 File). As expected, the CIAT 899 NF profile in the presence of dulcitol was similar to that obtained with 400 mM mannitol (Figure B in S7 File). Finally, both findings correlate with the synthesis and export of 35 different nodulation factors when CIAT 899 cultures were induced with dulcitol (Figure C in S7 File). Interestingly, most of these molecules were also identified upon induction with mannitol (29 out of 35), suggesting that both processes are carried out in a similar way. In conclusion, these results indicate that *R*. *tropici* CIAT 899 produces Nod factors in response to a non-ionic osmotic stress independently of the catabolism of the metabolite.

## Discussion

The osmolarity of the environment is one of the physical parameters that determines the capacity of organisms to proliferate in different habitats [38]. Our RNA-seq analysis revealed different genomic traits related to osmotic-stress tolerance in *R*. *tropici* CIAT 899 (Figs 1 and 2). Similar patterns of transcriptomic responses have been previously described for other bacteria in response to different abiotic-stresses. Common DEG encode proteins involved in the correct folding of proteins, chemotaxis, accumulation of organic osmolytes like glycerol, production of cyclic β-(1, 2)-glucans, transcription and translation or in the generation of energy (S2 File) [14,39–43].

*R*. *tropici* CIAT 899 harbours in its genome the genes necessary for the biosynthesis of trehalose, which could be used as a compatible solute in hyperosmotic conditions. However, transcriptomic data indicated that the *otsA* and *otsB* genes, involved in the biosynthesis of trehalose, are not differentially expressed when CIAT 899 was grown in the presence of mannitol. Fernández-Aunión et al., 2010 [44] have verified that although trehalose accumulation in CIAT899 is osmoregulated, the internal concentration of this osmolyte is not enough to explain high saline stress tolerance in *R*. *tropici* CIAT 899. The authors suggest that CIAT 899 should be accumulating other molecules such as cyclic β-1, 2 glucans in response to osmotic stress. In fact, the *ndvA* gene, which codes for the protein responsible for the synthesis and export of cyclic β-1, 2-glucans was up-regulated (Table 2). Moreover, the *thuAB* genes, involved in trehalose catabolism, were up-regulated in the presence of mannitol (Table 2).

To sum up, CIAT 899 does not accumulate trehalose in response to osmotic stress conditions but, instead, it degrades this carbohydrate. This fact could be explained because this osmolyte tends to accumulate during symbiosis, but it is toxic to plants [45]. To solve this problem, the bacterium would produce trehalose-degrading enzymes, but in turn, it would accumulate glycerol and cyclic glucans as compatible osmolytes in response to higher mannitol concentrations.

One of the biggest achievements in this study relies on the finding of the capacity of CIAT 899 to induce not only the expression of nodulation genes but also nitrogen fixation-associated genes under mannitol stressing conditions (Table 2). Besides, in contrast to previous transcriptomic reports with CIAT 899 grown in the presence of flavonoids or salt [14], not all the nodulation operons were up-regulated with mannitol (only the *nodA2nodEF* operon, the *nodA3*gene and the *nodD1* gene) (S3 File). However, these transcriptomic changes are enough to cause an increase in the production of biologically active NF under mannitol conditions (Fig 3, Table 3). Interestingly, the synthesis and export of these symbiotic molecules was also enhanced when this bacterium was grown with other non-metabolizable sugar (dulcitol), indicating that Nod factor production is specifically triggered by non-ionic osmotic stress, independently of the use of the metabolite that causes the stress (S7 File). On the other hand, in the same manner as with saline stress [17], the mannitol-mediated NF production and export is controlled directly by the NodD2 protein (Fig 3, Tables 2 and 3, S3 File). Besides, NodD2 is not only controlling the expression of nodulation genes but also the expression of other genes involved in protein folding and synthesis, motility, synthesis of polysaccharides and nitrogen fixation (Table 3, S3 File).

It has been well described that high concentrations of organic acids are secreted by plants to the rizosphere. In these conditions, CIAT 899 could be synthetising NF. In fact, RNA-seq assays from *R*. *leguminosarum* biovar *viciae* present in pea, alfalfa and sugar cane rhizospheres showed that genes coding for proteins related to sugar transport and catabolism are highly activated, including the same genes associated to mannitol catabolism (*mtlD* and *mtlE*) [46] also induced in the present study. Our hypothesis is that *R*. *tropici* CIAT 899 induces NF production in the presence of high concentrations of osmolytes to ensure the symbiotic interaction with legumes even in the absence of inducing flavonoids. This could be a strategy to overcome osmotic-stressing conditions. Interestingly, a recent study showed that in the symbiotic interaction between *Mesorhizobium loti* R7A and *Lotus japonicus*, NodD1 is activated during progression through the infection thread and this activation is required for a proficient symbiotic infection [4]. Thus, the mannitol-related induction of the nodulation genes in CIAT 899 (*nodF*, *nodE*, *nodA2*, *nodA3* and *nodD1*) could be indicating also that this activation may be taking place also during the development of the infection thread or even within the nodule, where high concentrations of osmolytes are expected. In this manner, the production of these symbiotic molecules in *R*. *tropici* CIAT 899 under high osmotic concentration could be occurring to support since the beginning to the end the symbiotic process. In these symbiotic environments, the activation of other genes important for late stages of the symbiosic process, such as some *fix*, *nif* or *fdx* genes, which encode proteins components of the nitrogenase machinery, could be explained (Table 2). Interestingly, the gene coding for the sigma factor 54 (YP_007336111.1), which has been widely described as responsible for *nif* and *fix* genes activation in microaerobiosis in several rhizobial strains within the nodule [47], showed a significant activation under mannitol conditions (Table 2). Thus, the activation of the whole nitrogen-fixing cluster under mannitol conditions could be a reflect of the transcriptomic changes that CIAT 899 endures in the legume nodule. Thereby, in the same manner as in the case of the NF synthesis and export, CIAT 899 could be ensuring nitrogen fixation inside the nodule not only by means of microaerobic conditions but also under high concentrations of osmolites present within the nodule. Nevertheless, further works are necessary to elucidate the full correlation between osmotic-stress and rhizobia-legume symbiosis.

## Supporting information

S1 File General features of the total sequenced and mapped reads. (DOCX)

S2 File Whole genome differential expression in *R*. *tropici* CIAT 899 cultures supplemented with 400 mM mannitol. Fold-change values are obtained in comparison with the cultures of the same strain without mannitol.
(XLSX)

S3 File Whole genome differential expression in the *nodD2* mutant cultures supplemented with 400 mM mannitol. Fold-change values are obtained in comparison with the cultures of the same strain without mannitol.
(XLSX)

S4 File Genes and primer sequences for *q*RT-PCR assays. Correlation degrees between RNA-Seq and *q*RT-PCR experiments. *q*RT-PCR and RNA-Seq fold-change values of 10 selected genes were represented in a graph to obtain the correlation degrees.
(XLSX)

S5 File Growth-rate curve of *R*. *tropici* CIAT 899 in TY medium at increasing concentrations of mannitol (55–1000 mM). (TIF)

S6 File Representation of the distribution of the genes of *R*. *tropici* CIAT 899 in its chromosome and different plasmids. In addition, the transcriptome results and distribution in the chromosome and plasmid of the wild-type strain under 400 mM mannitol are represented. DEG: Differentially expressed genes.
(TIF)

S7 File **A.** β-galactosidase activity of *R*. *tropici* CIAT 899 carrying plasmid pMP240 grown with 16.5 mM mannitol and induced with different dulcitol concentrations (0–483.5 mM). Expression data were individually compared with the expression without inducing molecules of the wild-type strain using the Mann-Whitney non-parametrical test. The asterisks (*) indicate a significant difference at the level α = 5%. **B.** Thin-layer chromatography analysis of Nod factors produced by *R*. *tropici* CIAT 899 grown under control and 383.5 mM dulcitol conditions (both containing 16.5 mM mannitol). **C.** Structures of Nod Factor synthesized in the presence or absence of 345 mM dulcitol (supplemented with 55 mM manitol) by *Rhizobium tropici* CIAT 899. NF structures are represented following the convention in Spaink, 1992 [48] that indicates the number of GlcNAc residues in the backbone (Roman numeral), the length and degree of unsaturation of the fatty acyl chain, and the other substituents, which are listed in the order in which they appear, moving clockwise from the fatty acid. Hex, Hexose; NMe, N-methyl group at glucosamine non-reducing residue; S, sulfate group at reducing glucosamine residue; Cb, carbamoyl group. *NF also synthetized in the presence of mannitol.
(TIF)
