# Supplementary material for: Osmotic stress activates nif and fix genes and induces the Rhizobium tropici CIAT 899 Nod factor production via NodD2 by up-regulation of the nodA2 operon and the nodA3 gene
Source: PLoS One. 2019 Mar 27;14(3):e0213298. doi: 10.1371/journal.pone.0213298 (PMC6436695; doi:10.1371/journal.pone.0213298)
Supplement: S1 File — (DOCX) [file pone.0213298.s001.docx]

**Supplementary file 1. General features of the total sequenced and mapped reads**.

**Results of the secondary analysis:**

**1-Total reads of each samples:**

| Sample name | Total reads | Mapped reads | % Mapped reads | High-Quality reads | % High-Quality reads |
| --- | --- | --- | --- | --- | --- |
| WT-Control-1 | 62.809.496 | 62.809.496 | 100 | 59.086.080 | 94.07 |
| WT-Control-2 | 26.949.354 | 26.949.354 | 100 | 25.146.294 | 93.31 |
| WT-Mannitol-1 | 65.092.162 | 65.092.162 | 100 | 59.464.042 | 91.35 |
| WT-Mannitol-2 | 57.223.022 | 57.223.022 | 100 | 52.627.962 | 91.97 |
| D2-Control-1 | 51.216.904 | 51.216.904 | 100 | 46.959.120 | 91.69 |
| D2-Control-2 | 55.279.252 | 55.279.252 | 100 | 51.006.678 | 92.27 |
| D2-Mannitol-1 | 51.239.636 | 51.239.636 | 100 | 45.311.622 | 88.43 |
| D2-Mannitol-2 | 60.297.422 | 60.297.422 | 100 | 55.502.862 | 92.05 |

**2-Quality read controls: GC content.** Distribution of GC content on mapped reads. A normal distribution around 45-65% is expected. The mapped reads presented an appropriate distribution of GC content.


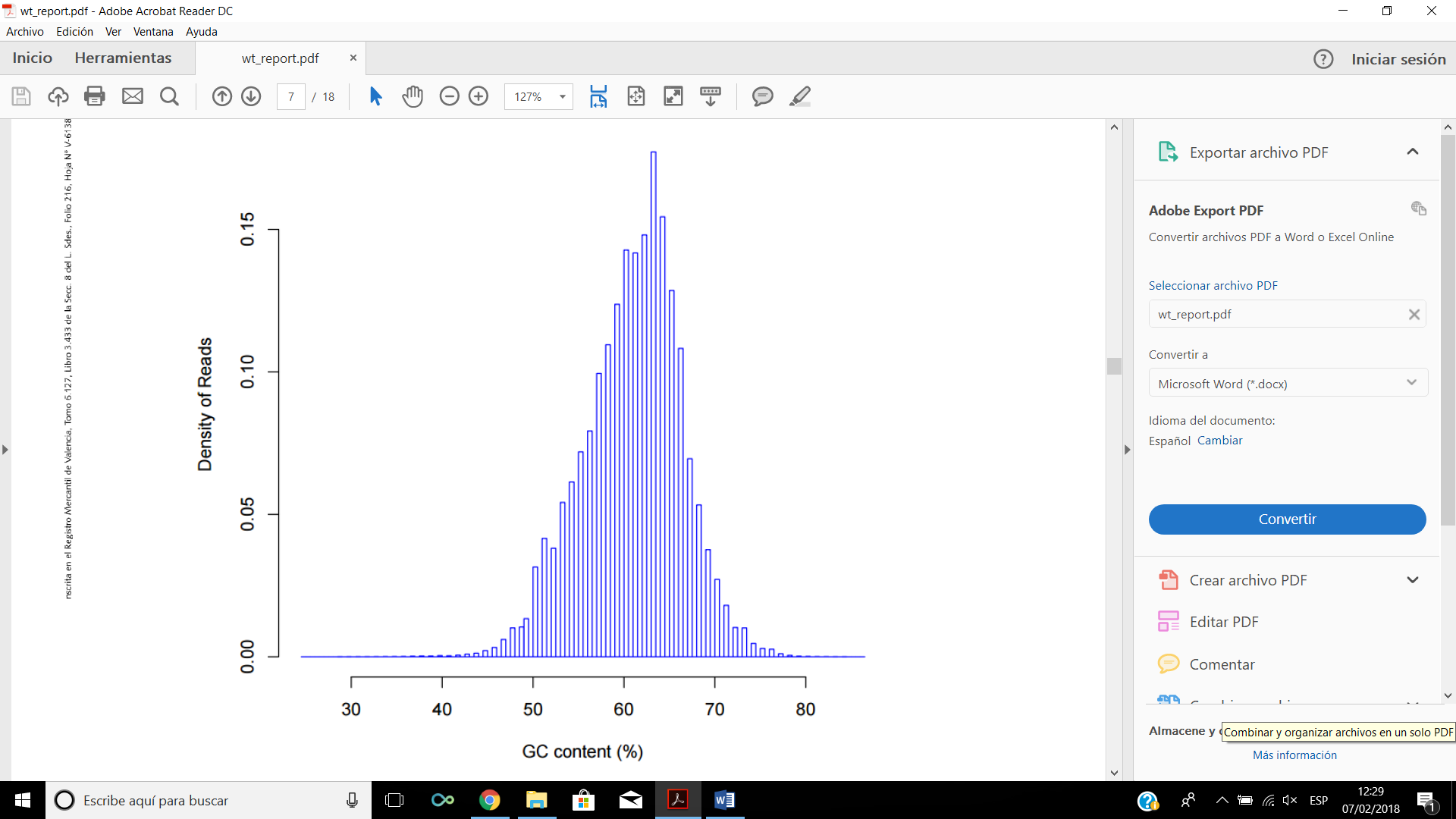


**3. Quality read controls: Duplicate distribution**. Common distribution of duplicates in a RNA-Seq experiment shows a small number of reads with high levels of duplicates and a high number of reads with low levels of duplicates. All samples presented optimal values of duplicate distributions.


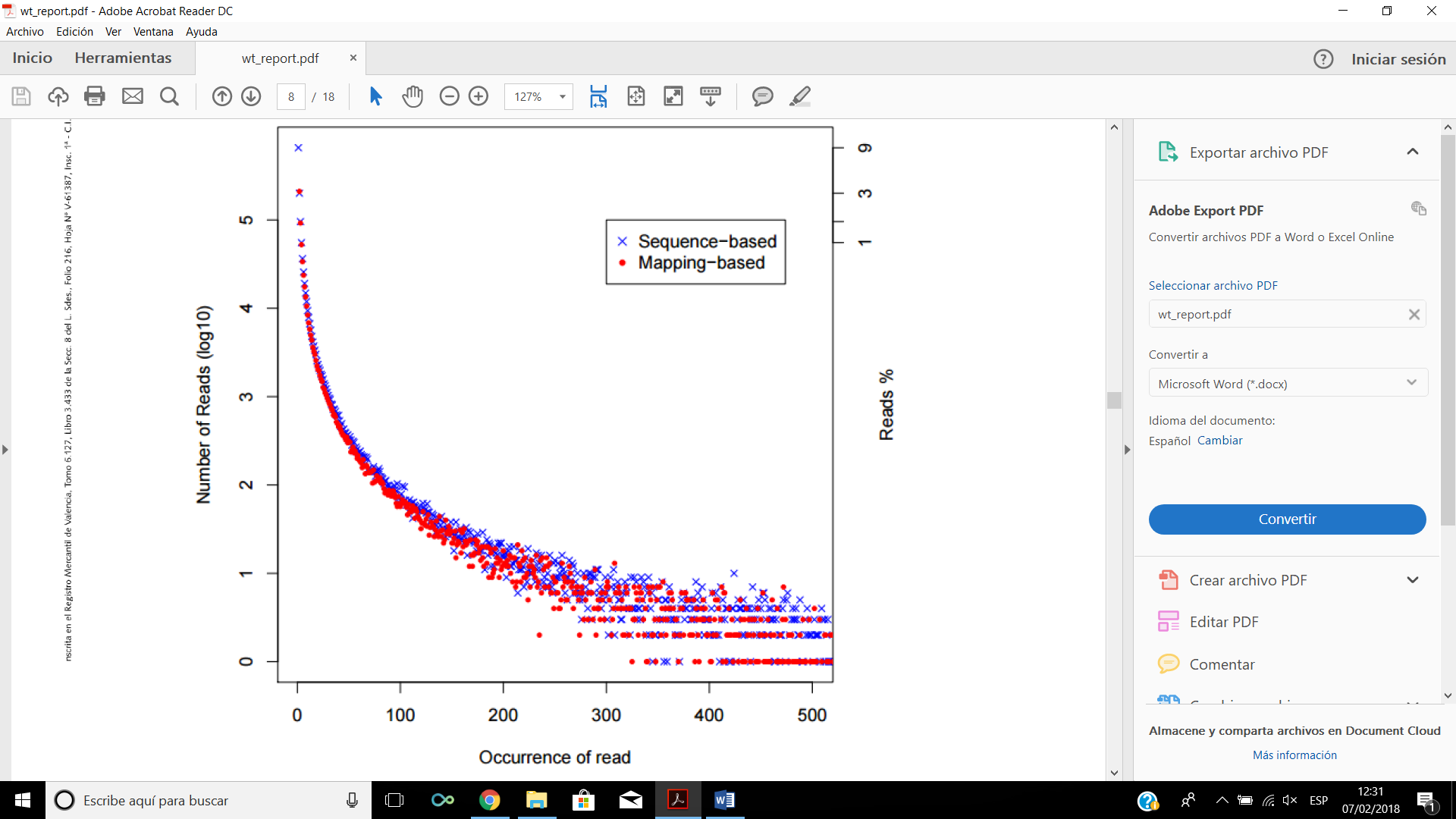


**Results of the tertiary analysis:**

**Normalization:** normalization is needed to avoid statistical deviations due to differences in library sizes. Each sample was compared withits corresponding control sample.

Number of reads per gene for controland mannitol samples of the wild-type strain **before** normalization.


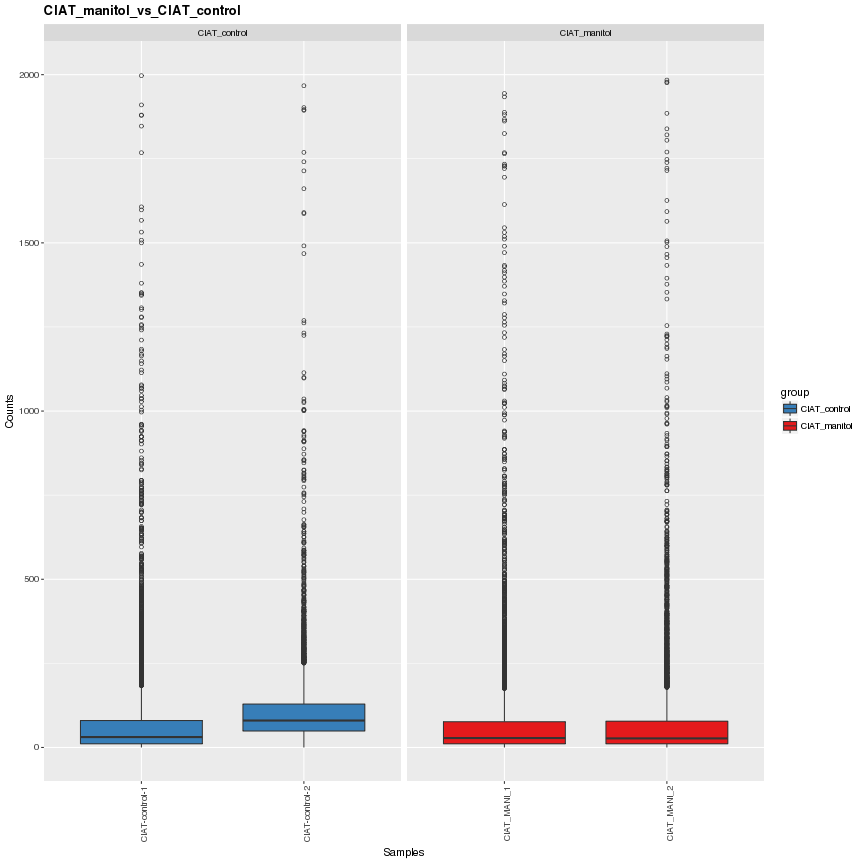


**number of reads per gene**

**WT-Control-1 WT-Control-2 WT-Mannitol-1 WT-Mannitol-2**

Number of reads per gene for control and mannitol samples of the wild-type strain **after** normalization.
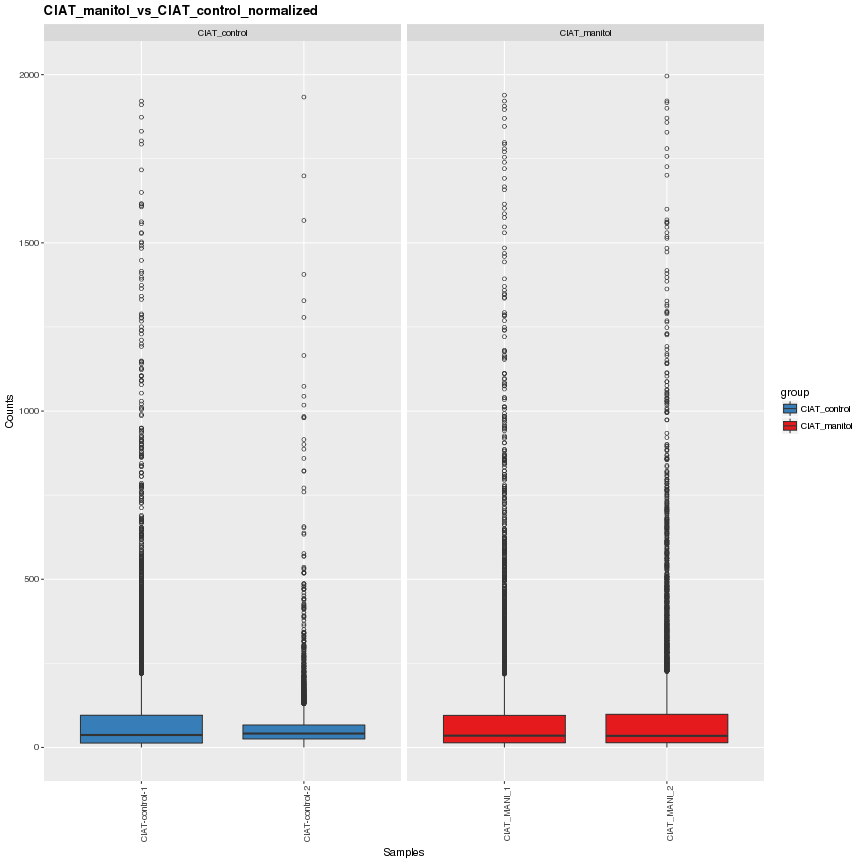


**number of reads per gene**

**WT-Control-1 WT-Control-2 WT-Mannitol-1 WT-Mannitol-2**

Number of reads per gene for the wild-type control and *nodD2* mutant mannitol samples **before** normalization.

**
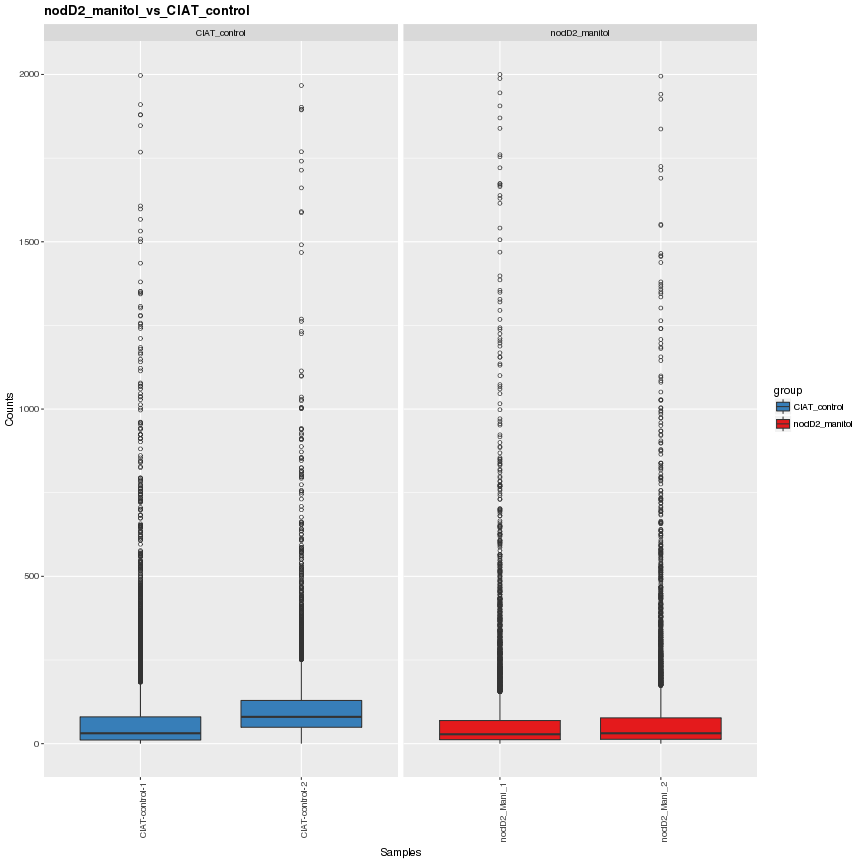
**

**number of reads per gene**

**WT-Control-1 WT-Control-2 D2-Mannitol-1 D2-Mannitol-2**

Number of reads per gene for the wild-type control and *nodD2* mutant mannitol samples **after** normalization.

**number of reads per gene**


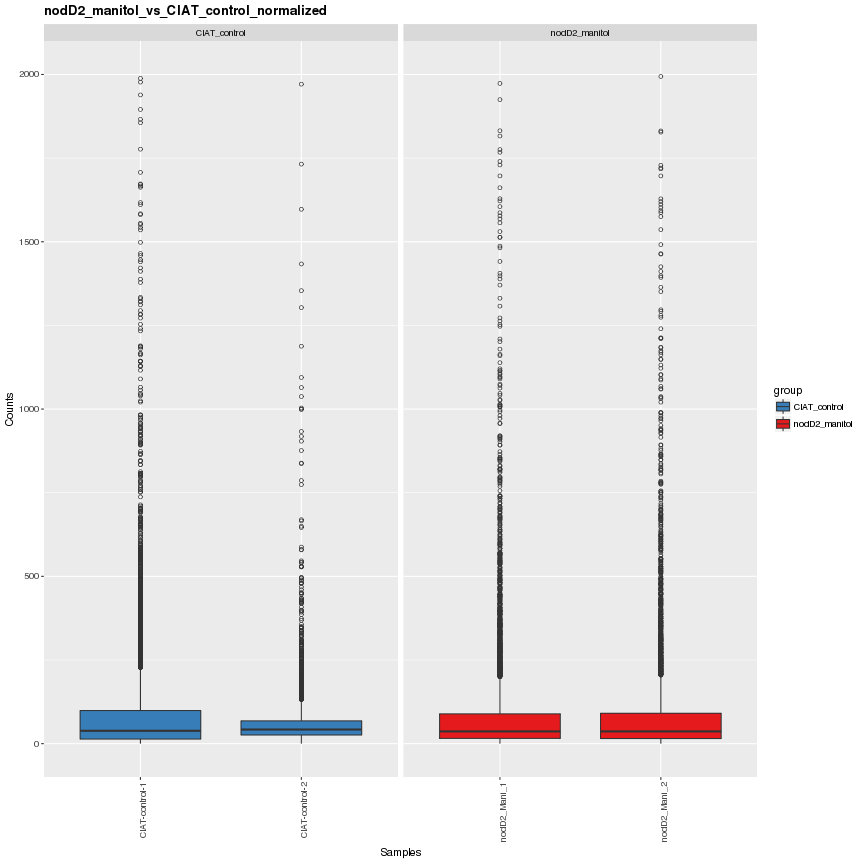


**WT-Control-1 WT-Control-2 D2-Mannitol-1 D2-Mannitol-2**
