# Supplementary figures and images for: Osmotic stress activates nif and fix genes and induces the Rhizobium tropici CIAT 899 Nod factor production via NodD2 by up-regulation of the nodA2 operon and the nodA3 gene
Source: PLoS One. 2019 Mar 27;14(3):e0213298. doi: 10.1371/journal.pone.0213298 (PMC6436695; doi:10.1371/journal.pone.0213298)

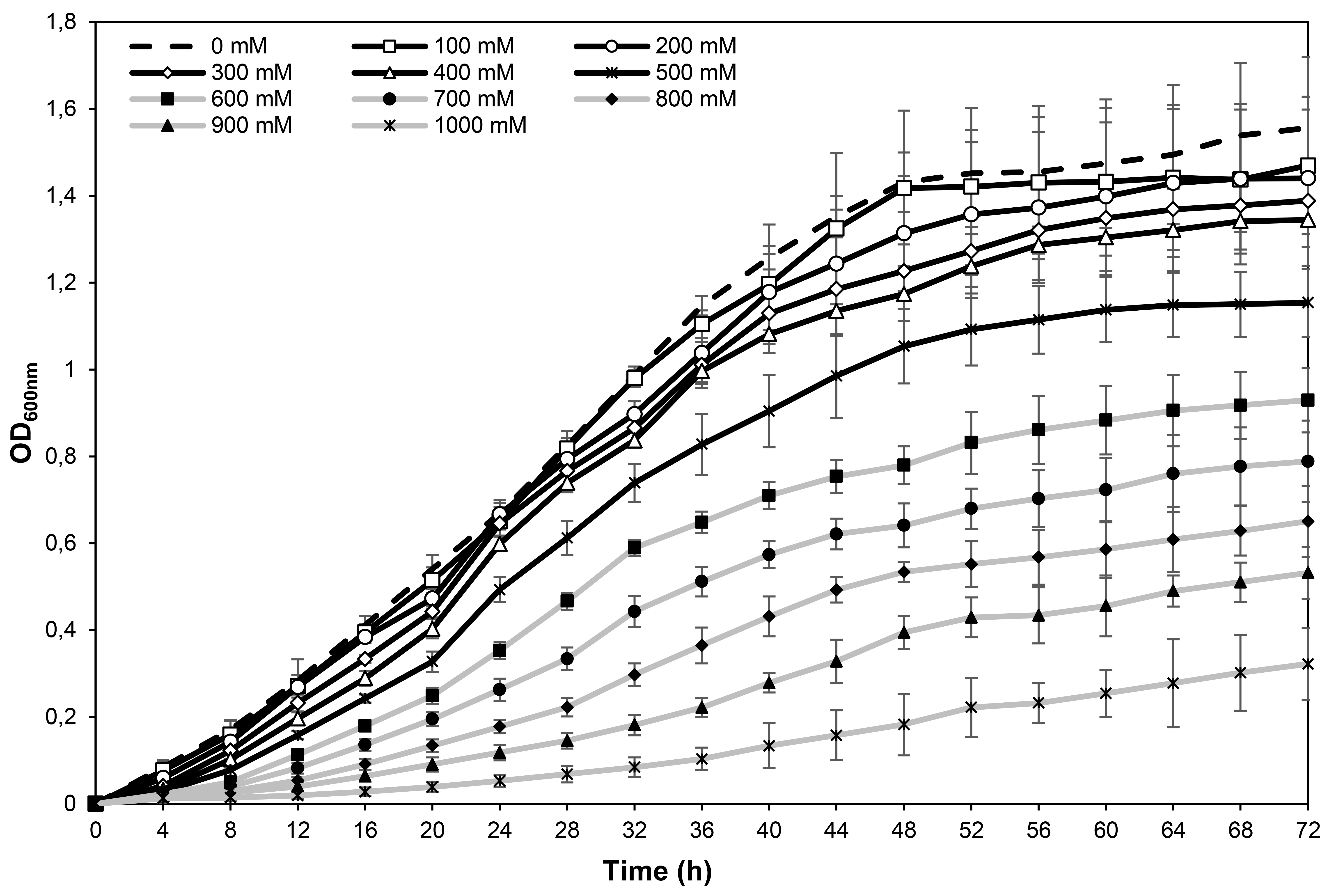

Supplement: S5 File — (TIF) [file pone.0213298.s005.tif]

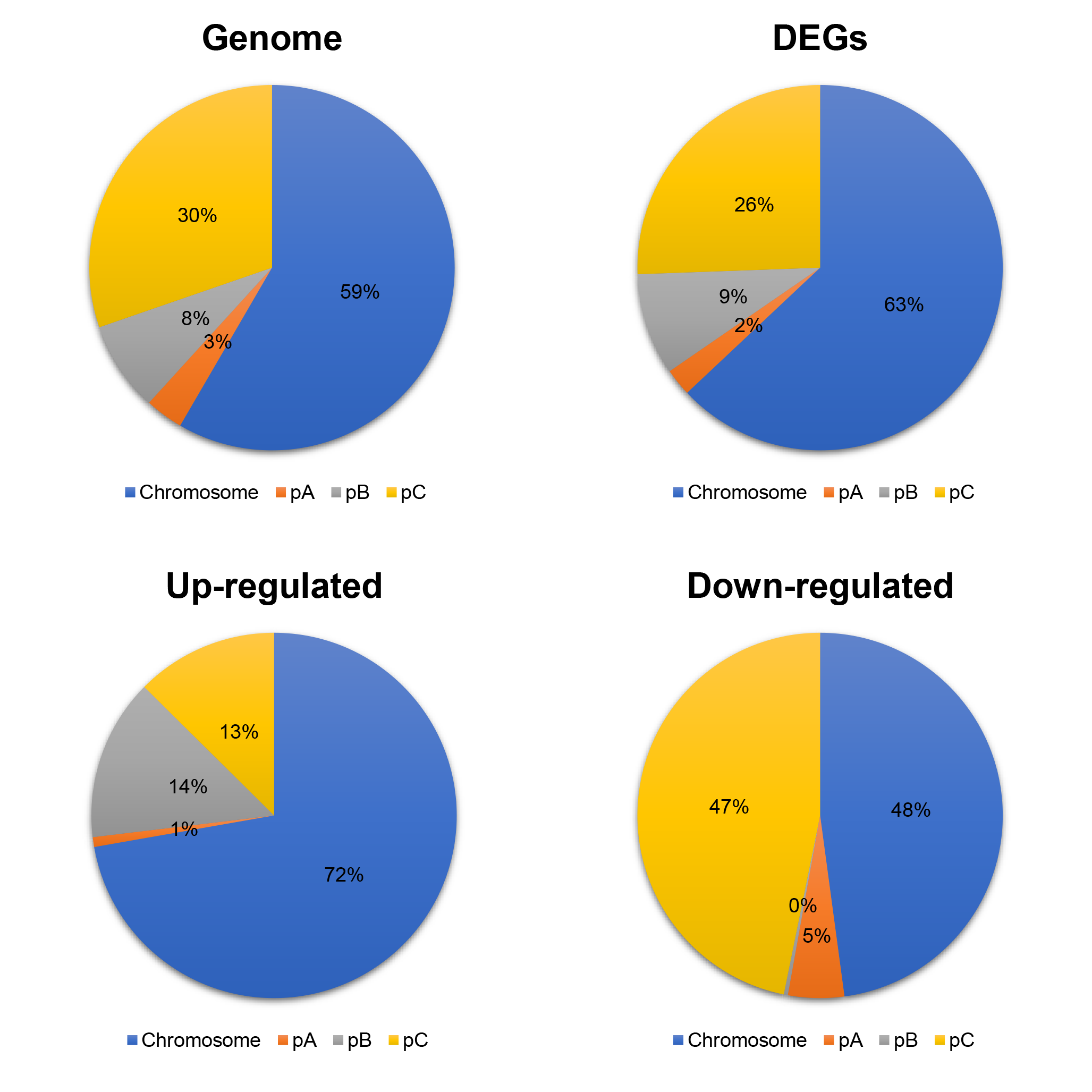

Supplement: S6 File — In addition, the transcriptome results and distribution in the chromosome and plasmid of the wild-type strain under 400 mM mannitol are represented. DEG: Differentially expressed genes. (TIF) [file pone.0213298.s006.tif]

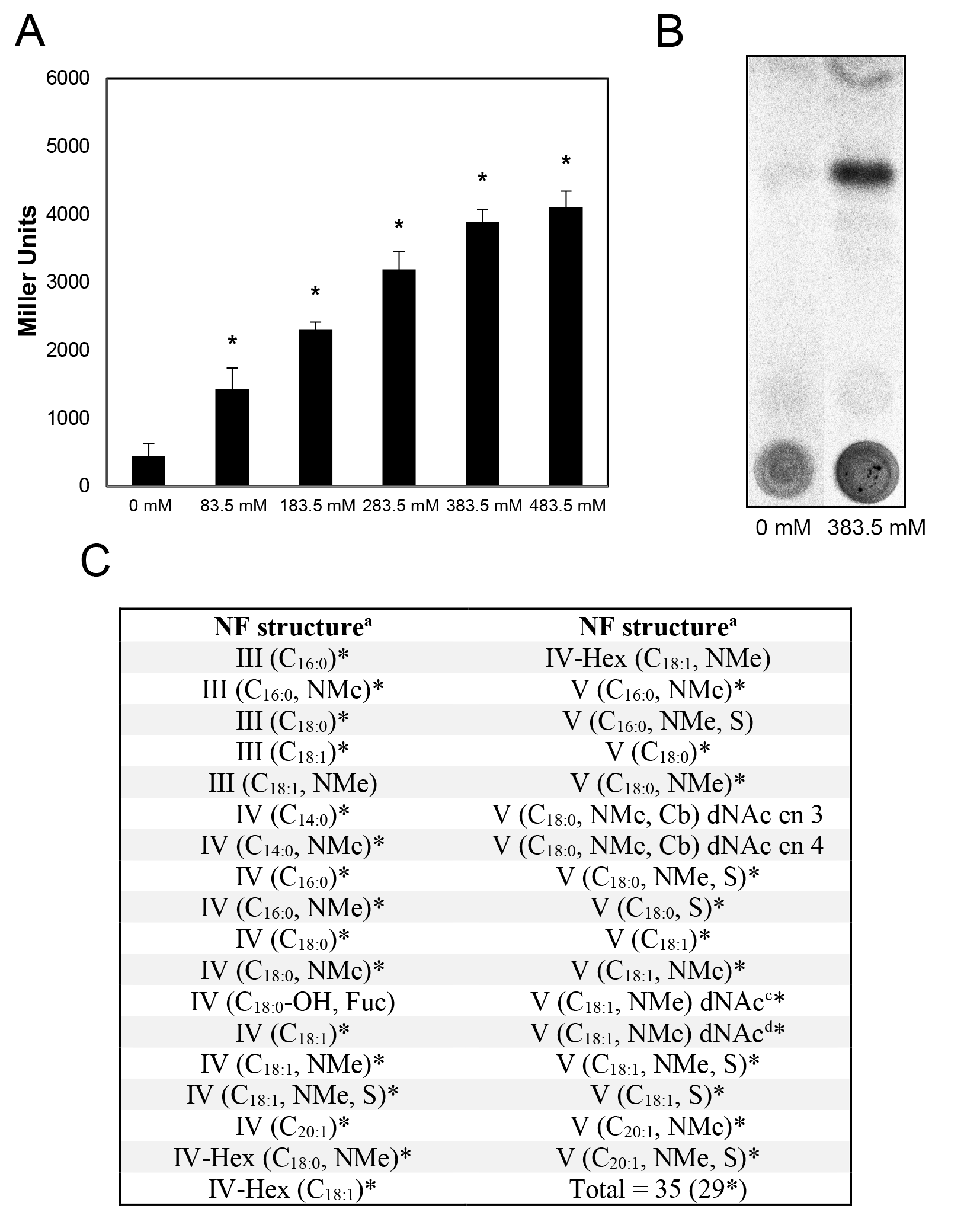

Supplement: S7 File — A. β-galactosidase activity of R. tropici CIAT 899 carrying plasmid pMP240 grown with 16.5 mM mannitol and induced with different dulcitol concentrations (0–483.5 mM). Expression data were individually compared with the expression without inducing molecules of the wild-type strain using the Mann-Whitney non-parametrical test. The asterisks (*) indicate a significant difference at the level α = 5%. B. Thin-layer chromatography analysis of Nod factors produced by R. tropici CIAT 899 grown under control and 383.5 mM dulcitol conditions (both containing 16.5 mM mannitol). C. Structures of Nod Factor synthesized in the presence or absence of 345 mM dulcitol (supplemented with 55 mM manitol) by Rhizobium tropici CIAT 899. NF structures are represented following the convention in Spaink, 1992 [48] that indicates the number of GlcNAc residues in the backbone (Roman numeral), the length and degree of unsaturation of the fatty acyl chain, and the other substituents, which are listed in the order in which they appear, moving clockwise from the fatty acid. Hex, Hexose; NMe, N-methyl group at glucosamine non-reducing residue; S, sulfate group at reducing glucosamine residue; Cb, carbamoyl group. *NF also synthetized in the presence of mannitol. (TIF) [file pone.0213298.s007.tif]
